# MST1 Regulates Neuronal Cell Death via JNK/Casp3 Signaling Pathway in HFD Mouse Brain and HT22 Cells

**DOI:** 10.3390/ijms20102504

**Published:** 2019-05-21

**Authors:** Mehtab Khan, Bart P. F. Rutten, Myeong Ok Kim

**Affiliations:** 1 Division of Life Science and Applied Life Science (BK21 Plus), College of Natural Sciences, Gyeongsang National University, Jinju 52828, Korea; mehtabneuro@gnu.ac.kr; 2 Department of Psychiatry and Neuropsychology, School for Mental Health and Neuroscience (MHeNS), Faculty of Health, Medicine and Life Sciences, Maastricht University, European Graduate School of Neuroscience (EURON), Maastricht, The Netherlands; b.rutten@maastrichtuniversity.nl

**Keywords:** high-fat diet, ROS, MST1, JNK, apoptosis, neurodegeneration

## Abstract

Oxidative stress has been considered as the main mediator in neurodegenerative diseases. A high-fat diet (HFD) and metabolic diseases result in oxidative stress generation, leading to various neurodegenerative diseases via molecular mechanisms that remain largely unknown. Protein kinases play an important role in the homeostasis between cell survival and cell apoptosis. The mammalian sterile 20-like kinase-1 (MST1) protein kinase plays an important role in cellular apoptosis in different organ systems, including the central nervous system. In this study, we evaluated the MST1/c-Jun N-terminal kinase (JNK) dependent oxidative damage mediated cognitive dysfunction in HFD-fed mice and stress-induced hippocampal HT22 (mice hippocampal) cells. Our Western blot and immunofluorescence results indicate that HFD and stress-induced hippocampal HT22 cells activate MST1/JNK/Caspase-3 (Casp-3) signaling, which regulates neuronal cell apoptosis and beta-amyloid-cleaving enzyme (BACE1) expression and leads to impaired cognition. Moreover, MST1 expression inhibition by shRNA significantly reduced JNK/Casp-3 signaling. Our in vivo and in vitro experiments mimicking metabolic stress, such as a high-fat diet, hyperglycemia, and an inflammatory response, determined that MST1 plays a key regulatory role in neuronal cell death and cognition, suggesting that MST1 could be a potential therapeutic target for numerous neurodegenerative diseases.

## 1. Introduction

Metabolic syndrome is a collection of evolving disorders that are characterized by insulin resistance, impaired glucose regulation, dyslipidemia, obesity, diabetes, and hypertension. Metabolic disorders are linked with brain disorders, including aging, Alzheimer’s disease, Parkinson’s disease, and other neurodegenerative dementias [1,2,3,4]. In recent years, attempts have been made to investigate metabolic dysfunction in terms of impaired brain insulin signaling, high triglyceride and cholesterol concentrations, and mitochondrial dysfunction, which collectively lead to endoplasmic reticulum stress in neurons and to cognitive disorders [5,6,7,8]. Argente-Arizon et al. demonstrated that metabolic disease is associated with either peripheral or central inflammation [9]. At the central level, the release of TNF-alpha, IL-1beta, and IL-6 cytokines increases insulin resistance, decreases neuronal plasticity and synapse formation, and increases amyloid beta (Aβ) formation [10,11,12]. Several studies have reported that metabolic syndrome results in the accumulation of reactive oxygen species (ROS), elevated oxidative stress, and neuroinflammation, leading to impaired biological processes [13,14,15]. Oxidative stress also elicits signaling pathways within the cells, leading to the modulation of gene expression and transcriptional activation, such as for the gene-encoding c-Jun, a transcriptional activator of NF-κB. Moreover, oxidative stress activates several stress-inducible protein kinases, such as extracellular signal-regulated protein kinase, c-Jun N-terminal kinase, p38, and sterile stress kinase 20-like protein kinase [16,17,18].

Mammalian sterile 20-like kinase 1 (MST1) is a serine-threonine kinase that constitutes a core component of the Hippo signaling pathway [19,20]. The MST family of kinases consists of MST1, MST2, MST3, and MST4 [21]. MST1 is reported to play important roles in the regulation of various biological effects, such as the response to oxidative stress and cell apoptosis regulation [22]. Several studies have reported that oxidative stress results in the activation of MST1, which further induces neuronal and neuroglia cell death [23,24,25]. A knockdown and overexpression analysis of CST-1, the ortholog of MST1 in *Caenorhabditis elegans*, showed effects on life span and aging, while genetic deletion experiments showed neuroprotection by attenuating neuronal apoptosis and an inflammatory response [26,27]. During apoptosis, MST1 is cleaved by caspases (Casp), which further activate other stress kinases mediated by p38 and c-Jun N-terminal Kinase (JNK) [28,29]. Tamagno et al. found that during oxidative stress beta-amyloid-cleaving enzyme (BACE1) activity is upregulated, which further results in the accumulation of amyloid beta and requires the activation of the c-Jun N-terminal kinase pathway [30]. Moreover, JNK inhibition studies have revealed the suppression of Casp-cleaved MST1 during apoptosis [31]. In the present study, we sought to examine the potential role of stress kinases MST1 and JNK in the regulation of neuronal cell apoptosis and their role in the regulation of BACE1 expression, which leads to impaired synaptic plasticity. Mimicking metabolic stress, such as a high-fat diet, hyperglycemia, and the inflammatory response, our in vivo and in vitro experiments determined the key regulatory effects of MST1 on synaptic plasticity and amyloid pathology.

## 2. Results

### 2.1. MST1 Mediates Oxidative Stress in HFD Mice Brain and HT22 Cells

**Figure 1:**
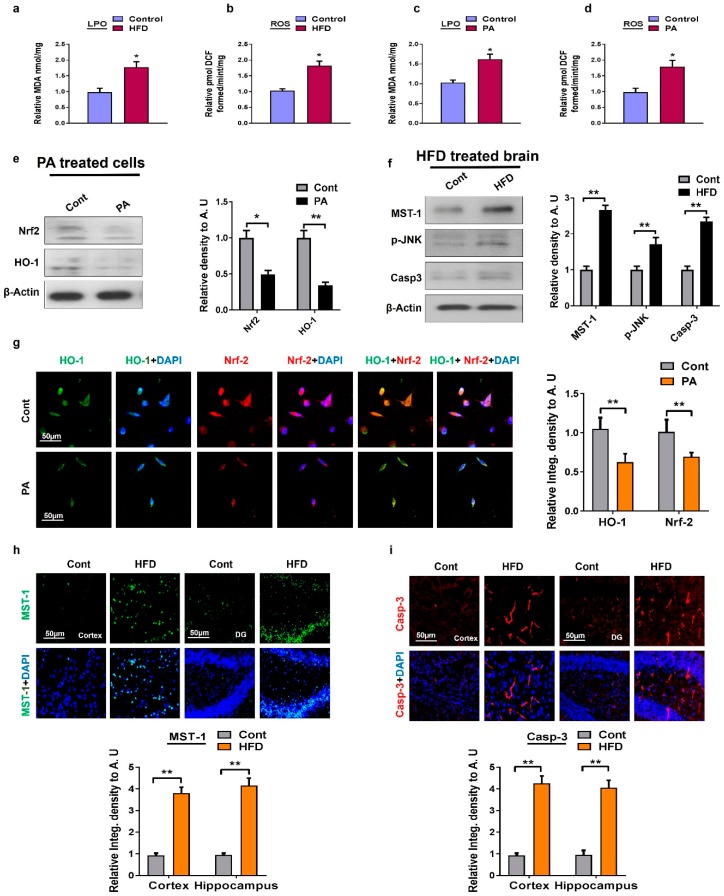
Metabolic dysregulation induced oxidative stress both in in vivo and in vitro models: (**a**,**b**) The histogram showing the results of lipid peroxidation (LPO) and reactive oxygen species (ROS) levels in the high-fat diet (HFD) mice model; (**c**,**d**) a histogram represent the results of LPO and ROS levels in palmitic acid treated HT22 cells; (**e**) shown are the Western blot results of nuclear factor-2 erythroid-2 (Nrf-2) and hemeoxygenase-1 (HO-1) along with respective histograms in the palmitic acid-treated HT22 cells. β-Actin was used as a loading control; (**f**) shown are the Western blot results of mammalian sterile 20-like kinase-1 (MST1), phosphor-c-Jun N-terminal Kinase (p-JNK), and Caspase-3 along with respective histograms in brain homogenates of HFD-fed mice and the normal control group. β-Actin was used as a loading control; (**g**) representative images of immunofluorescence staining of colocalization of Nrf2/HO-1 in palmitic acid-treated cells; (**h**,**i**) immunofluorescence staining images of MST1 and Casp-3 in mice cortex and the hippocampus region. *n* = 12 mice/group. The data are shown here as a mean ± SEM. * *p* < 0.05, ** *p* < 0.01.

Oxidative stress is linked to a broad range of pathological disorders and several age-dependent disorders, such as neurodegenerative diseases [32,33]. In order to determine whether MST1 might mediate oxidative stress, we quantified oxidative stress in terms of reactive oxygen species (ROS) and lipid peroxidation (LPO) in the high-fat diet (HFD)-fed mice brain and HT22 cells treated with palmitic acid. For this purpose, we performed the respective ROS and LPO assays. The ROS and LPO assay results demonstrated that HFD-fed mice and palmitic acid-treated cells significantly increased oxidative stress (Figure 1a–d). Similarly, our Western blot and immunofluorescence results of palmitic-acid-treated HT22 cells show a significant suppression of the nuclear factor-2 erythroid-2 (Nrf-2) and hemeoxygenase-1 (HO-1) protein expressions (Figure 1e,g). Mitochondrial damage is considered as the potential target of lipotoxicity. Therefore, we conducted another experiment to observe MST1-mediated cell death in a p-JNK/Casp-3 dependent manner in HFD-fed mice. Compared to the control mice, our in vivo immunofluorescence analyses revealed that HFD-treated mice significantly activated MST1 both in the cortex and hippocampus regions. The immunofluorescence results were further confirmed by Western blot analyses. From these results, we can conclude that MST1 enhances the activity of the p-JNK level and its downstream neurodegenerative apoptotic marker, Casp-3, both in the cortex and hippocampal regions of HFD-fed mice (Figure 1f,h,i).

### 2.2. MST1 Regulates JNK/Akt Signaling Pathway in HT22 Cells Treated with High Glucose/Palmitic Acid/IL-1β Cytokines

**Figure 2:**
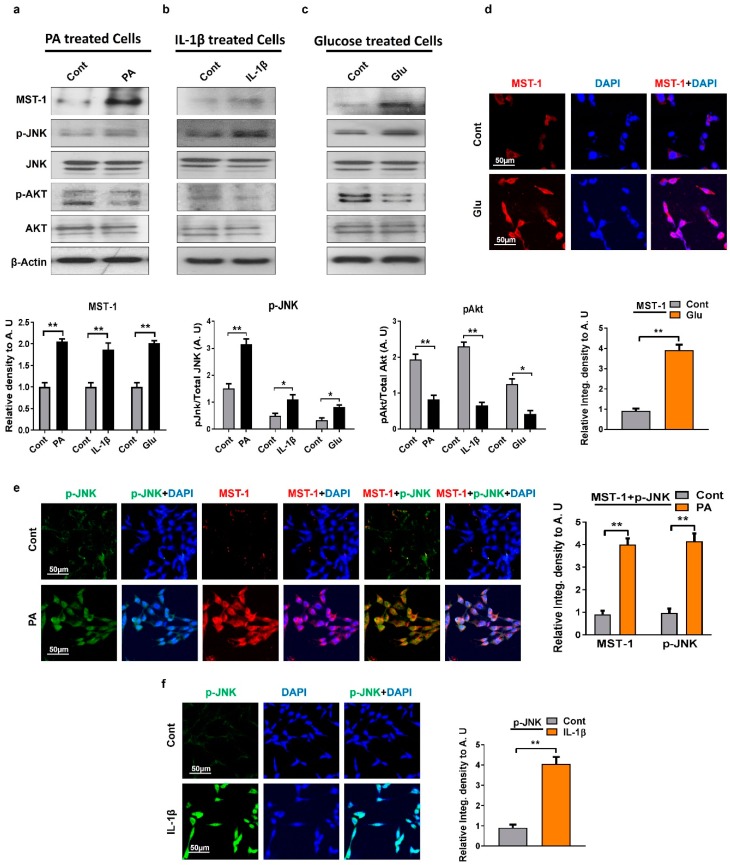
Metabolic dysfunctions regulate the expression level of MST1, p-JNK, and p-AKT in HT22 cells: (**a**–**c**) Western blot analysis of MST1, p-JNK, and protein kinase B (p-AKT) in palmitic acid, IL-1β, and glucose treated HT22 cells. The bands were quantified using ImageJ software, and the differences are depicted in the respective histogram. β-actin was used as a loading control. (**d**) Representative images of immunofluorescence staining showing MST1 expression in glucose-treated cells; (**e**) double immunofluorescence images of p-JNK and MST1 in palmitic acid treated HT22 cells; (**f**) representative immunofluorescence results of p-JNK expression in IL-1β treated HT22 cells. The data are shown here as a mean ± SEM. * *p* < 0.05, ** *p* < 0.01.

The c-Jun N-terminal kinase (JNK) is a member of the Mitogen-activated protein kinase (MAPK) family involved in cell proliferation, differentiation, and apoptosis. Studies have shown that MST1 is regulated by the stress kinase JNK that plays a critical role in the feedback regulation of the MST1 proapoptotic signaling cascade [34,35]. In order to examine the regulatory effect of MST1 on JNK signaling, mouse hippocampal HT22 cells were exposed to various metabolic stresses, such as chronic glucose, palmitic acid, and inflammatory interleukin 1-β (IL-1β) cytokines. The immunoblot and immunofluorescence results revealed that different models of metabolic stress (high glucose exposure, palmitic acid, or inflammatory cytokines) significantly upregulate and induce the phosphorylation of c-Jun N-terminal kinase and MST1 expression compared to untreated mouse hippocampal neuronal HT22 cells (Figure 2a–f). Various studies suggest a negative feedback mechanism between MST1 and Protein kinase B (AKT) [36]. We also checked the p-Akt protein marker through Western blot which is an important marker of the Phosphoinositide 3-kinase (PI3K)–AKT survival pathway. Our Western blot result showed a lower level of p-AKT expression compared to the untreated mouse hippocampal neuronal HT22 cells. In summary, our results suggest that oxidative metabolic stresses lead to an activation of the JNK-dependent MST1 stress kinase and an inhibition of p-AKT signaling.

### 2.3. MST1 Induces Neuronal Cell Apoptosis in HT22 Cells

**Figure 3:**
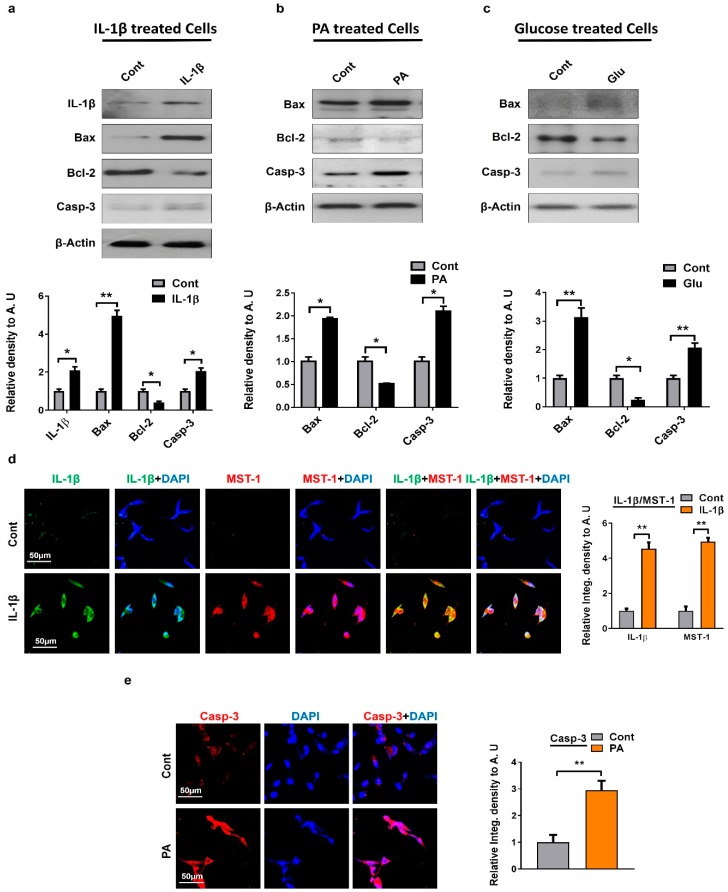
Metabolic deregulation induced apoptotic cell death in in vitro models: (**a**–**c**) Shown are the Western blot results of apoptotic markers Bax Bcl-2 can Cleaved-Casp-3 in IL-1β, palmitic acid, and glucose treated cells HT22 cells. β-actin was used as a loading control. For protein band quantification ImageJ software was used. One-way ANOVA followed by post-hoc analysis was used for statistical analysis. The density values were expressed in arbitrary units (AUs) as the mean ± SEM; (**d**) given are the representative images of double immunofluorescence staining of IL-1β and MST1 in IL-1β treated HT22 cells; (**e**) confocal microscopic results of Caspase-3 expression in palmitic acid treated HT22 cells. The data are expressed as the mean ± SEM. * *p* < 0.05, ** *p* < 0.01.

We sought to find out if MST1 stimulates mitochondria-mediated apoptosis. For that we checked whether the activation of MST1 is associated with neuronal cell apoptosis; we treated mouse hippocampal neuronal HT22 cells with a number of stress-inducing agents, such as high glucose treatment, palmitic acid, and inflammatory cytokines. In the mitochondrial apoptotic pathway, the proapoptotic B-cell lymphoma 2-associated X (Bax) and antiapoptotic B-cell lymphoma 2 (Bcl-2) are the main regulators [37]. Bcl-2 prevents neuronal cell death, while Bax enhances apoptosis by opening the mitochondrial permeability transition pores (MPTP), which subsequently release cytochrome c [38]. Similarly, IL1β and glucose triggers the opening of the MPTP, by action of a mitochondrial uncoupling agent, or by inhibition of the electron transport chain [39,40]. We conducted Western blot analyses for Bax, Bcl-2, and Cleaved-Casp-3. Our results show an increased expression of proapoptotic markers Bax and Cleaved-Casp-3 and a decline in antiapoptotic marker Bcl-2’s level in cells treated with stress-inducing agents when compared to untreated cells (Figure 3a–c). We also confirmed this through in vitro immunofluorescence assays showing a Cleaved-Casp-3 overexpression in palmitic-acid-treated cells compared to untreated normal cells (Figure 3e). IL-1β, a pro-inflammatory cytokine, plays an important role in the development of inflammatory responses and cell death in the brain against various CNS insults, such as acute injury and neurodegenerative diseases [41]. Progress in elucidating MST1 signaling and inflammatory stress in the regulation of cell death is still not clear and much remains to be learned. For that reason, we performed double immunofluorescence staining between IL-1β and MST1 in HT22 cells treated with IL-1β inflammatory cytokines. The result shows a high expression of both IL1-β and MST1 in cells treated with cytokines compared to untreated cells (Figure 3d). Altogether these findings highlight that MST1 is the key regulator of mitochondrial apoptosis.

### 2.4. MST1 Downregulation Attenuates JNK/Casp3 Signaling in HT22 Cells Treated with High Glucose/Palmitic Acid/IL-1β Cytokines

**Figure 4:**
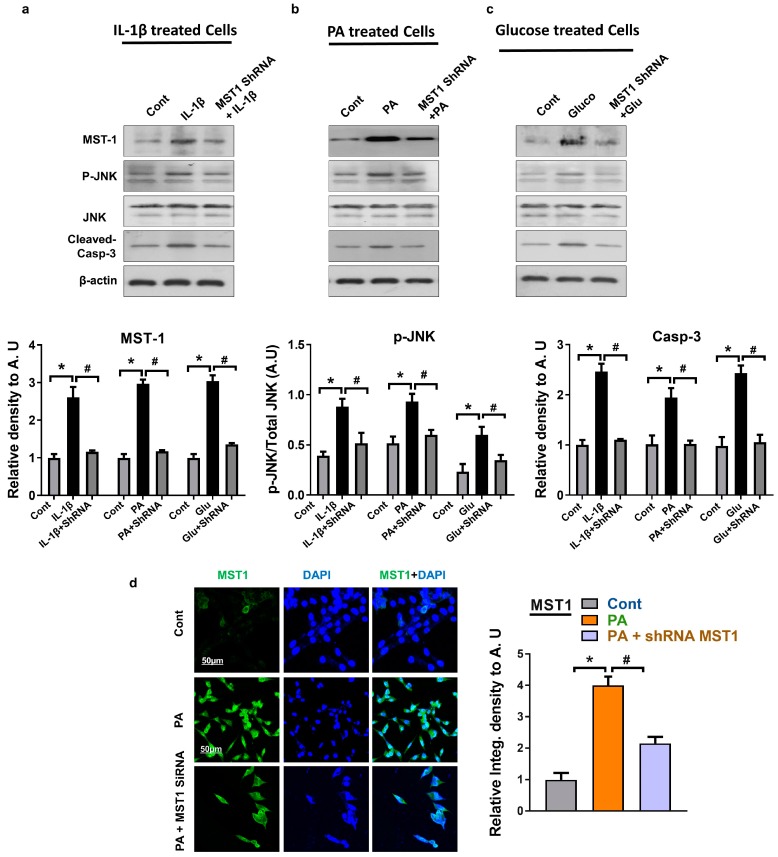
Effect of shRNA MST1 on JNK/Casp3 in HT22 cells: (**a**–**c**) Shown are the Western blot results of MST1, p-JNK, and Cleaved-Casp-3 in IL-1β, palmitic acid, and glucose treated cells HT22 cells. β-actin was used as a loading control. For protein band quantification ImageJ software was used. One-way ANOVA followed by post-hoc analysis was used for statistical analysis. The density values were expressed in arbitrary units (AUs) as the mean ± SEM; (**d**) given are the representative images of immunofluorescence staining MST1 in PA-treated HT22 cells; the data are expressed as the mean ± SEM. * Significantly different from the control group, and # Significantly different from the stress-induced group; * *p* < 0.05, # *p* < 0.05.

In order to clarify the potential role of MST1 in JNK/Casp-3 activation, we inhibited the MST1 levels in HT22 cells using transfection with shRNA MST1. We confirmed the downregulation of JNK and Cleaved-Casp-3 levels through immunoblot and immunofluorescence analysis in cells transfected with shRNA MST1 compared to cells treated with metabolic stressors (Figure 4a–d). The results revealed that shRNA MST1 downregulates MST1 expression, p-JNK, and the Cleaved-Casp-3 expression level compared to cells exposed to various metabolic stresses, such as chronic glucose, palmitic acid, and IL-1β inflammatory cytokines. The MST1 ShRNA results revealed that it did not completely abolish the MST1 level as compared to the control group, however, it moderately reduced the MST1 level. These results suggest that during a stress condition MST1 activates the JNK/Casp3 signaling pathway and enhances neuronal apoptosis.

### 2.5. MST1 Regulates BACE1, Aβ, and Synapsis in HFD Mice Brain and HT22 Cells

**Figure 5:**
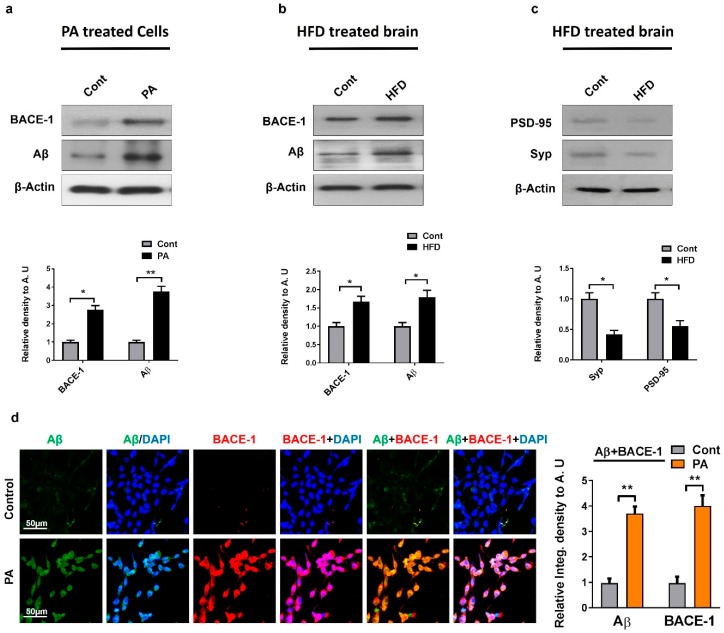
Metabolic dysfunction induced AD-like pathology both in in vivo and in vitro model: (**a**,**b**) The expression levels of Aβ and BACE-1 were assessed by Western blot in palmitic acid-treated HT22 cells, and in HFD mice. β-actin was used as a loading control. For protein band quantification Image J software was used. One-way ANOVA followed by post-hoc analysis was used for statistical analysis. The density values were expressed in arbitrary units (AUs) as the mean ± SEM; (**c**) the expression level of neuronal synapse proteins (both pre-synapse, i.e., Synaptophysin and post-synapse density protein 95, i.e., PSD95) along with their respective histograms were analyzed through Western blot in the HFD-fed mice brain; (**d**) given are the representative images of double immunofluorescence staining of Aβ and BACE-1in Palmitic acid treated HT22 cells. The data are expressed as the mean ± SEM. * *p* < 0.05, ** *p* < 0.01.

Studies have shown that oxidative stress elevates BACE1 expression and amyloid beta production through the activation of the stress kinase JNK pathway [42]. We performed Western blot analyses for BACE1 and Aβ in HFD-fed mice brain and mouse hippocampal neuronal HT22 cells treated with palmitic acid. We observed a significant increase in BACE1 expression and Aβ in the HFD-fed mice and stress-induced cells compared to the untreated control groups (Figure 5a,b). This effect was further confirmed by confocal microscopy, which showed an increased BACE1 and Aβ expression in HT22 cells exposed to palmitic acid (Figure 5d). Notably, oxidative stress along with Aβ deposition also leads to neuronal dysfunction, cell death, and a loss of synaptic connections [43]. To further evaluate the synaptic integrity, we quantified the expression of the presynaptic vesicle membrane synaptophysin (SYP) and the postsynaptic density protein 95 (PSD95) in HFD-fed mice brains. The Western blot analysis revealed a significant decrease in both the pre- and post-synaptic protein levels in HFD-fed mice compared with the control, indicating stress-induced synaptic dysfunction (Figure 5c).

## 3. Discussion

In the present study, we have unveiled a critical regulatory role of MST1, its counterpart JNK, and its activation of downstream signaling under various metabolic stress conditions. Herein, we present in vitro and in vivo evidence that metabolic stress-mediated ROS activates the MST1/JNK signaling pathway, executes neuronal apoptosis, and markedly increases the Aβ-producing enzyme BACE1, Aβ production, and synaptic loss. The main observations of the present study are the activation of MST1/JNK/Casp and amyloid pathology in HFD-fed mice and HT22 cells exposed to stress-inducing agents.

Existing literature demonstrates that alteration in metabolic processes leads to the formation of ROS and the induction of pro-inflammatory proteins, which leads to neurodegeneration and memory impairment. Oxidative stress directly affects mitochondrial function, as it is the major intracellular source of ROS [44,45,46]. Similarly, the main transcription factor regulators, such as HO-1 and Nrf-2, involved in antioxidation are downregulated in metabolic-dysfunction-mediated neurodegenerative disorder [47]. Our result also showed that HFD-fed mice and HT22 cells treated with metabolic stress-inducing agent palmitic acid resulted in oxidative stress via lipid peroxidation and ROS. Notably, Western blot results indicated that metabolic stress downregulates the Nrf-2 and HO-1 expressions. Previous studies have shown that stress kinase MST1 is highly upregulated due to the induction of JNK signaling and Casp-3-mediated cleavage in cells exposed to chronic glucose, palmitic acid, or upon exposure to acute oxidative stress from hydrogen peroxide [36]. We also confirm high MST1 expression in a JNK/Casp-3 dependent manner via both immunofluorescence and Western blot analyses in HFD-fed mice compared to normal chow-fed mice.

Studies have shown that oxidative stress triggers the MST1/JNK cascades associated with the apoptotic process [48,49]. Ura et al. [31] reported that JNK regulates MST1 activity via a feedback mechanism and results in the promotion of apoptosis. In agreement with previous studies, we also found a high expression of JNK and MST1 in HT22 cells treated with high glucose, palmitic acid, and IL-1beta pro-inflammatory cytokines. We next checked p-AKT, the main kinase in the PI3K survival pathway. Recent studies suggest possible crosstalk between MST1 and p-AKT and have shown a negative feedback mechanism under various stress conditions [50,51]. Our results also confirmed previously published data that acute stress decreases p-AKT activity which enables proapoptotic MST1 signaling to take place.

Oxidative stress results in mitochondrial membrane depolarization and cytochrome release from mitochondria to cytosol which further activates the Casp, thus leading to apoptosis [52,53,54]. MST1 also plays a key role in the increase of cell death [55]. Studies have shown the role of MST1 signaling in the mitochondrial pathway. An overexpression analysis of MST1 in mitochondria showed cleavage of Casp-9 and Casp-3, an initiation of proapoptotic Bax, and a decline in the BCL2 level [56,57]. We also analyzed the expression of Cleaved-Casp-3, Bax, and BCL2 in HT22 cells treated with high glucose, palmitic acid, and pro-inflammatory cytokine IL-1beta and concluded that under stress conditions MST1 is highly active and induces the mitochondrial-dependent apoptosis pathway in neuronal cells.

Studies have shown in both cellular and animal models that oxidative stress upregulates the expression and activity of BACE1 through the activation of the c-Jun N-terminal kinase/activator protein1 pathway [58,59,60,61]. Kim et al. reported that treating SK-N-MC cells with palmitic acid conjugated with bovine serum albumin (PA-BSA) results in an increased expression of BACE1 and Aβ production through the G protein-coupled receptor 40 (GPCR40) [62]. In correlation with previous studies, this study demonstrates for the first time that MST1/p-JNK signaling can also potentially stimulate the apoptotic pathway which ultimately leads to the upregulation of beta-amyloid-cleaving enzyme expression (BACE1), Aβ production, and synaptic marker loss. Our Western blot and immunofluorescence results reveal that HFD-fed mice brain cells and palmitic-acid-treated HT22 cells have a high expression of BACE1 and Aβ and a low expression of the synaptic marker in HFD-treated mice.

## 4. Materials and Methods

### 4.1. Chemicals

2′7′-dichlorodihydrofluorescein diacetate (DCFH-DA), 4′,6′-diamidino-2-phenylindole (DAPI), dimethyl sulfoxide (DMSO), d-Glucose (50 mM), and palmitic acid (PA) were purchased from Sigma Chemical Co. (St. Louis, MO, USA).

### 4.2. Animals and Drug Treatment

Wild-type male C57BL/6N mice of 8 weeks old (*n* = 45, weight 25–30 g) were purchased from Samtako Bio (Osan, Korea). All mice were housed for one week to acclimatize under standard laboratory conditions in a temperature of 23 ± 2 °C, relative humidity (60 ± 15%), and a 12-h light and dark cycle. The maintenance and treatment of mice were carried out in accordance with the Institutional Animal Care and Use Committee (IACUC) guidelines issued by the Division of Applied Life Science, Department of Biology, Gyeongsang National University, Republic of South Korea. The experimental techniques were approved (Approval number# GNU-170117-M0002, 17 January 2017) by the animal ethics committee (IACUC). The mice were randomly divided into two groups (*n* = 12 mice per group): Control group mice were treated and fed ad libitum with a standard chow diet (containing 21% protein, 48.8% carbohydrate, and 3% fat) and the HFD group were fed with a HFD (containing 26.2% protein, 26.3% carbohydrate, and 34.9% fat) (Cat.# D12492, Open Source Diet, Research Diets, Inc., New Brunswick, NJ 08901, USA). All the 8-week-old mice were fed with chow/HFD for 24 weeks and the mice were sacrificed at 32 weeks of age.

### 4.3. In Vitro Cell Culture and Drug Treatment

For in vitro cell line culturing, a mouse hippocampal (HT22) cell line was cultured in Dulbecco’s modified Eagle’s medium (DMEM) supplemented with 10% fetal bovine serum (FBS) and antibiotic/antimycotic (1%) in an incubator supplied with 5% CO_2_ and a temperature of 37 °C, as previously described [63,64]. After gaining 70% confluency, cells were treated with palmitic acid (150 μM), high glucose (50 mM) and IL-1beta cytokines (10 ng/mL) for 24 h.

### 4.4. Transfections

To knockdown MST1 in HT22 cells, cells were seeded until they attained 60% confluence; then, the culture media were removed, and the cells were washed with Opti-MEM media to remove traces of serum. Four shRNA constructs for Mouse MST1 (Origene, Rockville, MD, USA) with sequence TACCGTGGCGAGGTAGACGTTACAGAGTC, GCGCCTTGGTGCTTCACATCTCGACCTGG, TGTCATCTCCAACCAGGAATGTAACACGA, ATGCTACCACGGCTCAGGTGAACAGTATC was transiently transfected into HT22 cells using the Lipofectamine 3000 reagent (Thermo Fisher Scientific), according to the manufacturer’s protocols, and efficiently reduced the MST1 protein expression levels. Transfection media were replaced with normal cell culture media after 2 h. Cells were harvested and collected with a cell scraper or adherent cells on cover slips were fixed with a neutrally buffered paraformaldehyde (NBP) solution for 25 min prior to transfer to microscopic slides.

### 4.5. Western Blot Analysis

The Western blot analysis was conducted as previously performed in our lab [65,66]. First, the mice were euthanized via giving proper anesthesia and then sacrificed, brains were carefully collected, and, for freezing, tissues were kept on dry ice. Furthermore, the brains were homogenized carefully in PRO-PREP extraction solution. For the in vitro hippocampal neuronal HT22 cells, cells were collected in 1% phosphate buffer saline (PBS) and centrifuged followed by the removal of the supernatant. The remaining pellets were dissolved in PRO-PREP extraction solution, according to the manufacturer’s protocol (iNtRON Biotechnology, Burlington, NJ, USA), and were sonicated to make cell lysates. Both the brain homogenate and cell lysate were quantified with the Bio-Rad protein assay solution. A total of 20–30 μg protein was fractioned by SDS-PAGE gel followed by transferring proteins from gel to a polyvinylidene difluoride (PVDF) membrane. After transfer, the membranes were blocked in 5% BSA/skimmed milk and incubated overnight, with primary antibodies at 4 °C, washed with 1× tris-buffered saline (TBS) and tween (TBST), and reacted with secondary antibodies for 1–2 h. ECL (Amersham Pharmacia Biotech, Uppsala, Sweden) detection reagent was used for visualization, according to the manufacturer’s instructions. The densities of bands were quantified by using ImageJ software and graphs were generated via GraphPad Prism 6 software.

### 4.6. Antibodies

A list of the antibodies (primary and secondary) used in this study is provided in Table 1.

### 4.7. Tissue Collection and Sample Preparation for Morphological Study

As previously described [67], the mice were subjected to transcardial perfusion with ice-cold PBS followed by 4% neutrally buffered paraformaldehyde (NBP). After post fixing the brain in 4% NBP for 72 h, it was washed with PBS and then transferred to 20% sucrose solution for a further 48 h. The brain tissue was frozen in optimal cutting temperature (OCT) (Tissue-Teks O.C.T. Compound Medium, Sakura Finetek USA, Inc., Torrance, CA, USA) and sectioned into 14–16 μm sections in the coronal plane with a CM 3050S cryostat (Leica, Wetzlar, Germany). The sections were thaw mounted on Probe-On positively charged slides (Thermo Fisher Scientific Inc., Waltham, MA, USA).

### 4.8. Immunofluorescence

Immunofluorescence staining was performed as previously reported [68]. Briefly, the brain-tissue-containing slides and fixed cells were washed twice with 0.01 M PBS for 15 min followed by proteinase K and blocking solution. Primary antibodies were added and incubated overnight at 4 °C. After washing, FITC/TRITC conjugated secondary antibodies (Santa Cruz Biotechnology) were then applied at room temperature for 1–2 h. Slides were washed twice with PBS for 5 min. Next, 4′,6-diamidino-2-phenylindole (DAPI) was applied to stain the nucleus, and glass coverslips were mounted on the slides with mounting medium. Images were captured using a confocal laser scanning microscope (FluoView FV1000 Olympus, Japan). Image J software was used to analyze the integral optical density (IOD).

### 4.9. Oxidative Stress (ROS) Detection In Vivo and In Vitro

The ROS quantification assay in the brain homogenates was conducted as previously described, with slight modifications [69,70]. It is based on the oxidation of 2,7-dichlorodihydrofluorescein (DCFH) to 2,7-dichlorofluorescein (DCF). Brian homogenates were diluted at 1:20 with ice-cold Locke’s buffer to get a final 5 mg tissue/mL concentration. Then, the reaction mixture (1 mL), having Locke’s buffer of pH 7.4, 0.2 mL brain homogenate, and 10 mL of DCFH (5 mM), was incubated for 15 min at room temperature to allow the DCFH-DA to be incorporated into any membrane-bound vesicles, and the diacetate group was cleaved by esterases. After 30 min of further incubation, the conversion of DCFH-DA to the fluorescent product dichlorofluorescin (DCF) was measured using a spectrofluorometer with excitation at 484 nm and emission at 530 nm. The ROS formed was quantified from a DCF-standard curve, and the data were expressed as pmol DCF formed/min/mg protein. Similarly, an in vitro ROS assay was performed with slight modification, as previously described [71]. Briefly, HT22 cells were sub-cultured in 96-well plates in 200 μL DMEM that was supplemented with 10% FBS and 1% penicillin/streptomycin in every well. The cells were incubated for 24 h at 37 °C in a humidified incubator having 5% CO_2_. The next day, the media were replaced with fresh media that contained PA (150 µM), high Glucose (50 mM) and IL-1β (10 ng/mL) for an additional 30 min. Cells were exposed to DCFH (50 μM) and incubated for 30 min. The plates were then read in ApoTox-Glo™ (Promega, Madison, WI, USA) at 488/530 nm.

### 4.10. Lipid Peroxidation Assay

The LPO analysis was carried out as described previously [72,73]. In order to measure the lipid peroxidation for the determination of oxidative stress, free malondialdehyde (MDA), a marker of LPO, was measured in the brain homogenate of both the control and high-fat-diet-fed mice using a malondialdehyde colorimetric/fluorometric assay kit (Cat# K739-100 Bio Vision, BioVision, San Francesco, CA, USA) in accordance with the manufacturer’s protocol. For the in vitro analysis of LPO, HT22 cells were cultured in DMEM medium (200 µL/well) in 96-well plates followed by incubation for 24 hours in a humidified incubator (5% CO_2_, 37 °C). After incubation, PA (150 µM) was diluted in fresh DMEM medium to replace the previous one, and then the LPO analysis was conducted as mentioned for the in vivo experiment.

### 4.11. Statistical Analyses

For the Western blot analyses, the original X-ray films were scanned and analyzed via Image J software (National Institutes of Health, Bethesda, MD, USA). The integral optical density (IOD) of the morphological analysis was also performed by Image J software. The data were expressed as the mean ± SEM of triplicate wells for in vitro experiments and of 10–12 mice per group for in vivo experiments—these were representative of three independent experiments. Statistical analysis between groups was conducted by using GraphPad Prism6 software (San Diego, CA, USA) followed by a one-way analysis of variance (ANOVA) with Tukey’s post hoc test. Values of *p* < 0.05 were considered to be significant.

## 5. Conclusions

On the whole, our findings showed that stress kinases, MST1 and JNK, have potential roles in the regulation of neuronal cell death, beta-amyloid-cleaving enzyme level, and synaptic dysfunction. We suggest a more detailed and mechanistic approach to elucidate MST1 signaling in various neurological diseases and that inhibition of this signaling pathway may provide a new therapeutic avenue.

## Figures and Tables

**Table 1:** List of primary and secondary antibodies used in this study and their information.

Antibody	Catalog	Application (Conc.)	Host	Manufacturer
anti-β- actin	sc-47,778	WB (1:1000)	Mouse	Santa Cruz Biotech. (Dallas, TX, USA)
anti-Akt	sc-514032	WB (1:1000)	Mouse	Santa Cruz Biotech. (Dallas, TX, USA)
anti-BACE1	sc-33711	WB (1:1000)	Mouse	Santa Cruz Biotech. (Dallas, TX, USA)
anti-Aβ	sc-28365	WB/IF (1:1000/1:100)	Mouse	Santa Cruz Biotech. (Dallas, TX, USA)
anti-HO1	sc-136961	WB (1:1000)	Mouse	Santa Cruz Biotech. (Dallas, TX, USA)
anti-IL-1β	sc-32294	WB (1:1000)	Mouse	Santa Cruz Biotech. (Dallas, TX, USA)
anti-Nrf2	sc-722	WB (1:1000)	Mouse	Santa Cruz Biotech. (Dallas, TX, USA)
anti-p-JNK	sc-6254	WB (1:1000)	Mouse	Santa Cruz Biotech. (Dallas, TX, USA)
anti-Bax	sc-7480	WB (1:1000)	Mouse	Santa Cruz Biotech. (Dallas, TX, USA)
anti-PSD-95	sc-71,933	WB (1:1000)	Mouse	Santa Cruz Biotech. (Dallas, TX, USA)
anti-Bcl2	sc-7382	WB (1:1000)	Mouse	Santa Cruz Biotech. (Dallas, TX, USA)
anti-Syp	sc-365447	WB (1:1000)	Mouse	Santa Cruz Biotech. (Dallas, TX, USA)
anti-AKT	sc-5298	WB (1:1000)	Mouse	Santa Cruz Biotech. (Dallas, TX, USA)
Anti-JNK	sc-7345	WB (1:1000)	Mouse	Santa Cruz Biotech. (Dallas, TX, USA)
anti-MST1	# 3682S	WB/IF (1:1000/1:100)	Rabbit	Cell Signaling Tech. (Danvers, MA, USA)
anti-Cl-Caspase-3	#9664	WB (1:1000)	Rabbit	Cell Signaling Tech. (Danvers, MA, USA)

WB: Western blotting, and IF: Immunofluorescence.

## Abbreviations

BSA	bovine serum albumin
CNS	central nervous system
DAPI	4’,6-diamidino-2-phenylindole
ECL	Enhanced chemiluminescence
DCFDA	2′,7′-dichloroflourescin diacetate
DMEM	Dulbecco’s modified eagle’s medium
FBS	fetal bovine serum
FITC	Fluorescein isothiocyanate
HFD	High -Fat Diet
IL-1β	Interleukin 1 beta
LPO	Lipid peroxidation
MTT	3-(4,5-Dimethylthiazol-2-Yl)-2,5-Diphenyltetrazolium Bromide
MST1	mammalian Ste20-like kinases
NF-κB	nuclear factor kappa-light-chain-enhancer of activated B cells
PA	Palmitic acid
PBS	phosphate buffer saline
p-JNK	phospho c-jun N terminal kinase
ROS	reactive oxygen species
SDS-PAGE	sodium dodecyl sulfate poly acrylamide gel electrophoresis
TRITC	Tetramethyl rhodamine isothiocyanate
